# Herding or Hunting? Animal Exploitation at Jiangzhuang (Jiangsu, China) During the Liangzhu Period (3300–2300 BC)

**DOI:** 10.3390/ani14233461

**Published:** 2024-11-29

**Authors:** Yifei Liu, Yucheng Huang, Huiyuan Gan, Ningning Dong

**Affiliations:** 1 Nanjing Museum, Nanjing 210015, China; liuyifei981221@163.com; 2 Department of Cultural Relics and Museology, Fudan University, Shanghai 200443, China; 22307080060@m.fudan.edu.cn; 3 Jiangsu Provincial Institute of Cultural Relics and Archaeology, Nanjing 210016, China; ganhy1984@163.com

**Keywords:** zooarchaeology, Liangzhu, animal exploitation, Lower Yangtze Valley

## Abstract

The lower Yangtze Valley provides a rich context for studying the interplay between the origins and intensification of agriculture and socio-political complexity. While the adoption of agriculture was closely associated with the developing societal complexity in major core centres during the Liangzhu period (3300–2300 BC), smaller and peripheral sites, which were also integral parts of the regional social fabric, exhibited contrasting subsistence choices and varying societal development. By examining these locations, a better understanding of the core-hinterland relationships within the region’s complex social structure and agricultural background would be clarified. In this paper, we present a zooarchaeological study of the Jiangzhuang site, located on the northern periphery of the Liangzhu culture. The faunal assemblage is primarily composed of cervids with a small proportion of suids, indicating subsistence heavily relying on wild resources. The morphological variation in suids is likely a result of the co-existence of domesticated pigs, wild boar, feral pigs, and probably the hybrid of the three, unveiling a complex human-suid relationship. The intensive utilisation of cervid bones for tool production is also a distinctive feature. While the Jiangzhuang community might have been articulated with the Liangzhu centre through the circulation of ritual jades, the subsistence economy there remained largely self-sufficient. The economic independence in the peripheral region might have contributed to the increasing decentralisation in the late Liangzhu period, providing a plausible explanation for the collapse of Liangzhu society.

## Simple Summary

This zooarchaeological study, examining faunal remains recovered from the Jiangzhuang site, attempts to dissect the interplay between subsistence choices and societal development during the Liangzhu period (3300–2300 BC) in the Lower Yangtze Valley. We found that in this rural village peripheral to the Liangzhu core area, Jiangzhuang residents heavily relied on wild animal resources, among which cervids were intensively utilised. A variety of suids, including domesticated pigs, wild boar, feral pigs, and hybrids of the three, were also present, suggesting a complex human-suid relationship involving both husbandry and hunting. Subsistence at Jiangzhuang was self-sufficient, and its rejection of intensive agriculture was attributed to natural and social factors. The economic independence in the peripheral Liangzhu areas might have contributed to the collapse of Liangzhu society.

## 1. Introduction

The interplay between the origins and intensification of agriculture and the development of complex societies has been continuously explored by archaeologists for more than a century. In recent decades, the Lower Yangtze Valley, where archaeological discoveries suggest a twin process of agricultural intensification and the development of socio-political complexity [1,2,3], has provided a rich context for revisiting this question.

Liangzhu (3300–2300 BC), a late Neolithic culture in the Lower Yangtze Valley, is very likely the earliest state-level society in East Asia, supported by the discoveries of the fortified urban centre, advanced water management system, grand ritual altars, hierarchically organised cemeteries and exquisite jade crafts in its core area in Yuhang, Zhejiang Province [4,5,6,7,8]. This socio-politically complex society was founded on a subsistence economy embracing intensified paddy-field rice farming [3,9] and urban animal management reliant on pig domestication [10,11]. A closer investigation into the subsistence, however, illustrates a mosaic pattern across the wide distribution of the Liangzhu culture, which could be contextualised in the core-hinterland relationship [11]. Being a regional sovereign power, the Liangzhu culture covered a wide area, with its borders extending to the coastal areas in the east and to the north across the Yangtze River. While the discoveries of the ritual jade *cong* imply a uniform political and religious matrix across these interconnected sites under the Liangzhu’s control [12], subsistence in peripheries demonstrates contrasting scenarios from that in the Liangzhu core area. In the coastal area, which is the easternmost distribution of the Liangzhu culture, previous zooarchaeological studies at Daxie [13], Tashan [14], and Wuguishan [15] found that wild herbivores outnumber the domesticates, and marine fish form a considerable proportion of the assemblage. A similar pattern was found at river-side sites in the Lake Tai basin, such as Chuodun [16], Longnan [17], and Shaoqingshan [16], where wild resources were heavily exploited, and the domesticates were maintained at a small scale. As these sites were located adjacent to rivers, freshwater fish were exploited, forming a local strategy distinctive from the coastal subsistence [13]. The highly localised and adaptive subsistence strategies reveal a more intricate variety within the core-periphery dynamics, adding a layer of complexity to interpret the interplay between the subsistence economy and societal development.

It remains debatable whether the northern border of the Liangzhu culture extended north of the Yangtze River until the discovery of the Jiangzhuang site in Xinghua, Jiangsu Province. The Jiangzhuang site is the first Liangzhu settlement located north of the Yangtze River. The northern edge of Liangzhu, distant from the core region, had nuanced differences in climate and environment. In addition, it was the frontier zone that the Liangzhu, Dawenkou, and Longshan cultures encountered. Unlike Liangzhu’s eastern peripheries in coastal areas, where geography restricted their connection with other cultures, Jiangzhuang stood at the pivotal point where multiple cultural communities might have interacted. Both Dawenkou and Longshan were influential cultures in the Jianghuai region, particularly in the perspective of agriculture and jade/pottery production [18] (pp. 240–241). Against this background, it remains under-explored how local subsistence was shaped. The faunal analysis of the Kaizhuang site in Dongtai, Jiangsu Province, provides a glimpse of subsistence in this northern frontier despite the small sample size of the faunal assemblage excavated from the Liangzhu period. Based on 236 pieces of the identified specimens (including 85 pieces of tortoise shell fragments), it could be tentatively concluded that wild cervid hunting, together with small-scale pig management, was practised [19]. Further in-depth studies zooming on the regional varieties with more abundant data will enhance our comprehension of the dynamic interplay between subsistence choices and societal processes during the Liangzhu period. Therefore, in this paper, we present a zooarcheological study of the Jiangzhuang site and attempt to unveil various environmental, political, and economic factors that shaped local subsistence.

## 2. Materials and Methods

**Figure 1:**
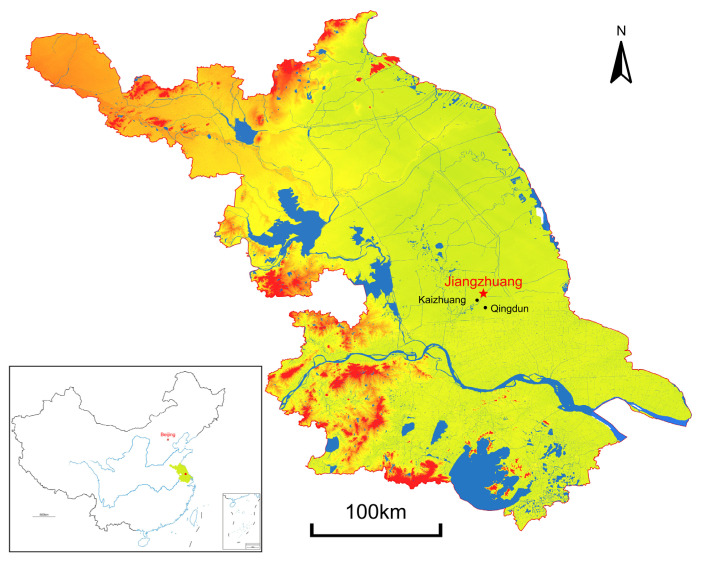
Map showing the location of the Jiangzhuang site.

The Jiangzhuang site, located between Xinghua and Dongtai in Jiangsu Province, lies on the southeastern part of the Lixiahe Plain, an area of low-lying terrain in the eastern region of the Lower Yangtze and Huai Rivers (Figure 1). The site is surrounded by rivers and streams, and wild resources are abundant in the surrounding environment. Carbon dating of human bones demonstrates site occupation between 3000 and 2400 BC, roughly contemporaneous to the Liangzhu period [20]. The Jiangzhuang site was accidentally found because of the waterway widening program. Archaeological excavations were conducted in 2011–2012 and 2015–2016 by the Archaeological Department of Nanjing Museum, covering an area of 20,000 m^2^. Burials, houses, pits, wells, and ditches were recovered, along with approximately 1200 relics, including jades, lithics, pottery, and bone tools [21].

A large number of ritual jades, such as *cong* and *bi*, were interred in the graves. This was the first time that such high-grade tombs of Liangzhu were found north of the Yangtze River. *Ding* (tripod), double-nosed *hu* (pot), jars, and *dou* (high-raised dish) were also discovered, representing a typical combination of pottery in Jiangzhuang [21]. The majority of human remains are well preserved, and the high proportion of young individuals with external skeletal injuries suggests that there might have been wars or violent conflicts during the Liangzhu period [22,23].

The Jiangzhuang site was the first large-scale settlement of the Liangzhu culture found to the north of the Yangtze River and the largest cemetery outside the Liangzhu core area. This corrects our misunderstanding that the distribution of the Liangzhu culture was confined to the southern reaches of the Yangtze River [21]. Pottery wares unearthed at the site display typological characteristics of local styles, whereas jades absorbed a large number of Liangzhu cultural factors [20]. Scholars believe that Jiangzhuang was likely the result of the integration of Liangzhu and the local culture [21]. Located in the northern frontier of the Liangzhu culture, the Jiangzhuang site was an important node for exchange between local culture in the eastern region of the Yangtze and Huai River, and the Liangzhu culture around the Tai Lake valley during the late Songze to Liangzhu periods [22].

A significant number of faunal remains were recovered from Jiangzhuang, providing new data for a comprehensive understanding of lifeways in the Liangzhu peripheral areas.

The faunal remains at Jiangzhuang were excavated from various contexts, including trial trenches, pits, wells, and burials dating to the late Liangzhu period [21,22]. Given that they were found to be fragmented and with no articulation with each other, most of the faunal remains were likely refused from daily life. The faunal assemblage was hand-collected. Sieving was not employed, owning to the time limitation of rescue excavation. The lack of sieving, however, might have resulted in the underrepresentation of smaller-sized fauna such as fish, one of the key food resources in the Lower Yangtze Valley, and younger individuals whose teeth and bones are relatively more fragile. We are cautious about the bias brought by the collecting method and consider their potential influences on data interpretation as necessary. Identification was performed at the Jiangnan Workstation, Nanjing Museum. Several published skeletal atlases [24,25,26] were referred to aid the on-site identification. Some specimens were compared to the reference collection at the Institute of Archaeological Science, Fudan University. The number of identified specimens (NISP) instead of MNI (Minimum Number of Individuals) counts was calculated to compare the proportion of each taxon, considering that many specimens were only identified at the family level. Measurements were recorded according to von den Driesch’s protocol [27].

Age profile, sex distribution and seasonality, body element distribution, and surface modification were examined to further understand past human-animal relationships. The age of the suids was estimated based on the eruption and wearing of the mandibular teeth [28,29]. Epiphyseal fusion pattern is also considered [28]. For ageing cervids, the mandible wear stage is utilised for consistency in the ageing method. Bowen’s method [30] for fallow deer (*Dama dama*) is used considering their similarity in body size with the Jiangzhuang cervids. Epiphyseal fusion information of cervids is recorded according to Carden’s method [31]. The presence or absence of antlers is useful for estimating sex and seasonality in most cervids [32,33,34]. The sex distribution and seasonality of milu and sika deer were reconstructed based on their crania and antlers. Bone surface modifications reveal human activities and taphonomic processes. Burning was recorded as present/absent. Observations of human and animal modifications were made with the naked eye and with the aid of a hand-held magnifier (Mobilux 12.5X, Eschenbach, Nuremberg, Germany).

## 3. Results

A total number of 6139 pieces of bone were collected, among which the NISP was 3945, including 202 specimens identified to the order level and 3675 specimens to the family level and below. The identified ratio is 64.3%, suggesting relatively good preservation. For the accuracy and consistency of the data, we conducted a subsequent analysis of animal bones that could be identified at the family level and below.

### 3.1. Taxon and Proportion

**Figure 2:**
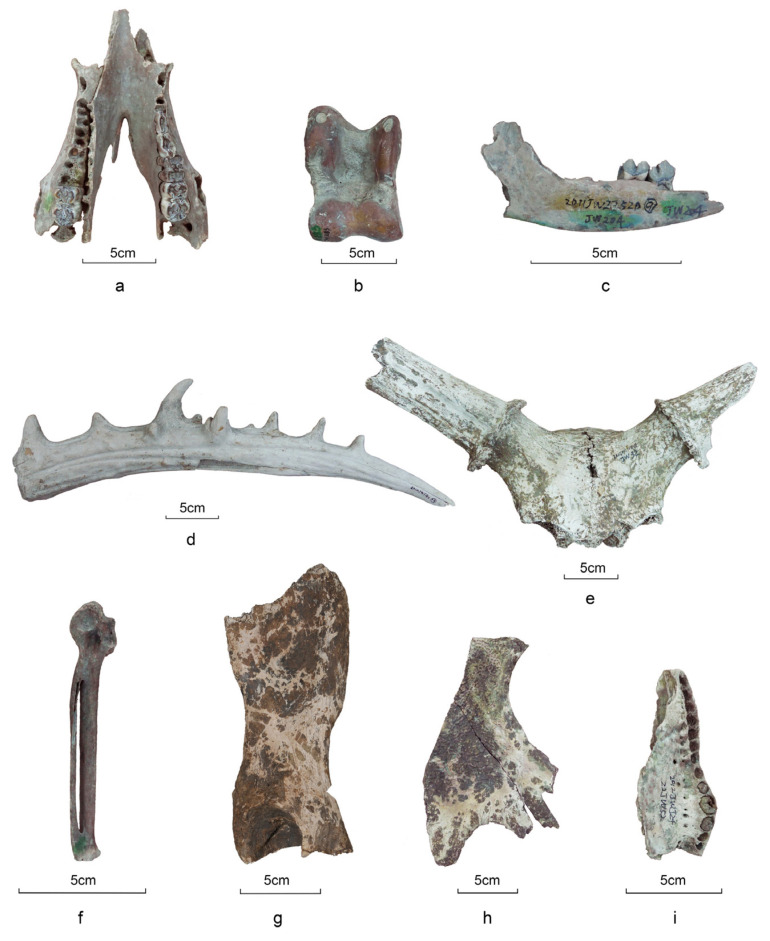
Faunal assemblage at Jiangzhuang: (**a**) pig mandible; (**b**) bovid astragalus; (**c**) dog mandible; (**d**) milu deer antler; (**e**) milu deer skull; (**f**) anser carpus; (**g**) tiger pelvis; (**h**) Yangtze giant softshell plastron; (**i**) Yangtze alligator maxilla.

**Figure 3:**
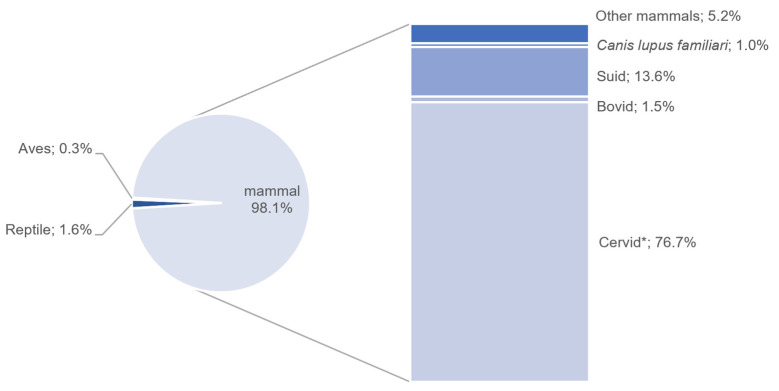
Percentage of animal faunal taxa (* including antlers).

A wide range of taxa was recovered at Jiangzhuang (Figure 2). In total, seven orders and ten families were identified (Table 1 and Figure 3), including mammals (NISP = 3869, 98.1%), reptiles (NISP = 63, 1.6%), and birds (NISP = 13, 0.3%). No fish were found, probably because the assemblage was not sieved at the time of collection.

Cervids constitute the largest proportion (NISP = 3026), including 827 antler pieces. Based on antler morphology, milu deer (*Elaphurus davidianus*, NISP = 51) and sika deer (*Cervus nippon*, NISP = 39) were identified. There are also small-sized deer, such as muntjac (*Muntiacus* sp., NISP = 6). For those who cannot further be identified to species, large-sized (n = 1376), medium-sized (n = 766) and small-sized (n = 57) cervids were identified regarding their body size.

**Figure 4:**
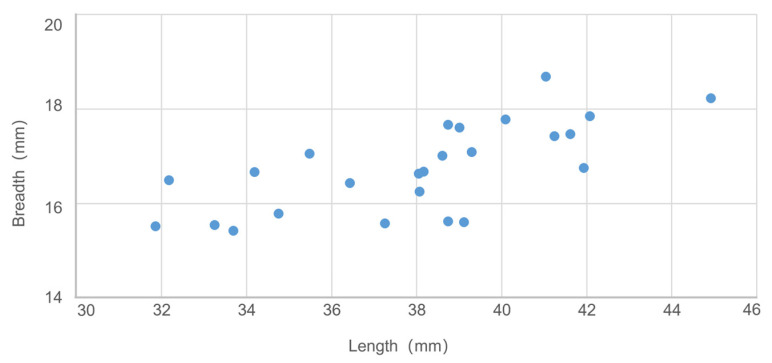
Distribution of lower M3 size of Jiangzhuang suids.

**Figure 5:**
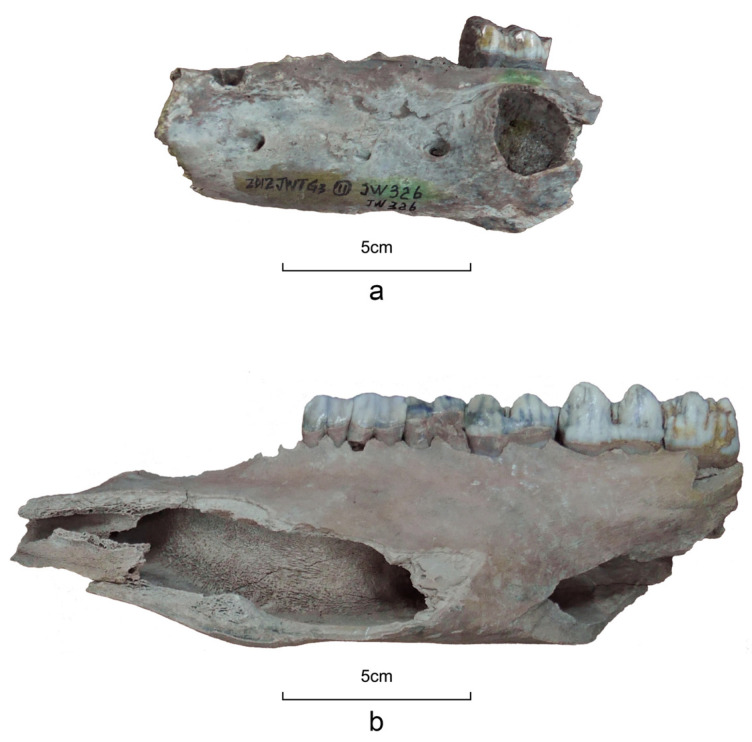
Comparison of the mandible from the (**a**) domesticated pig with a bone lesion caused by dentoalveolar abscess and (**b**) the wild boar.

Suids, including domesticated pig (*Sus scrofa domesticus*), wild boar (*Sus scrofa*), and morphologically ambiguous specimens, rank second in terms of bone quantity (NISP = 536). Measurements of the mandibular third molar (M3) reveal a span from 31 to 45 mm, with seven specimens exceeding 40 mm and two surpassing 42 mm (Table S1, Figure 4). According to data sets collected in China [35] (pp. 408–411), domesticated pigs have smaller molars with lengths usually less than 40 mm, whereas in wild boars, the length tends to be longer than 40 mm. Given that this criterion is affected by exogenous factors, including molar wearing related to age, size variation between regional suid groups, nutritional status, and so forth [36,37], we did not use it alone to identify suid status at the individual level. Some mandibles exhibit varying degrees of dentition distortion and bone lesions caused by dentoalveolar abscesses (Figure 5). The wide distribution of molar size in Jiangzhuang suids, along with their oral pathologies and mandible morphology, indicates the co-existence of probably domesticated pigs, wild boars, feral pigs, and hybrids of the three.

There are recoveries of bovid remains (NISP = 61). Cattle (*Bos taurus*) were introduced into the Central Plains in China approximately 4500 years ago [38]. It remains uncertain whether these animals had arrived in the Lower Yangtze Valley by the late Liangzhu period. Some researchers suggest that bovid remains discovered at contemporaneous sites might be from *Bubalus mephistopheles*, a local buffalo species [39,40]. More evidence is required to confirm the bovid species recovered from Jiangzhuang.

There are dog (*Canis lupus familiari*) remains excavated at the Jiangzhuang site (NISP = 39). In addition, one tiger (*Panthera tigris*), one civet (Viverridae), and one small-sized carnivore were identified.

Apart from mammals, reptiles and birds were also recovered. Among the reptiles, one Yangtze alligator (*Alligator sinensis*) and one carapace of a large soft-shelled turtle, probably from either *Pelochelys cantorii* or *Rafetus swinhoei,* were discovered. These two taxa were reported at several Neolithic sites in the Lower Yangtze Valley [41,42,43]. The avian remains, including five wild geese (*Anser* sp.), one swan (*Cygnus* sp.), and two cranes (Gruidae), were recovered.

### 3.2. Age Profile

**Figure 6:**
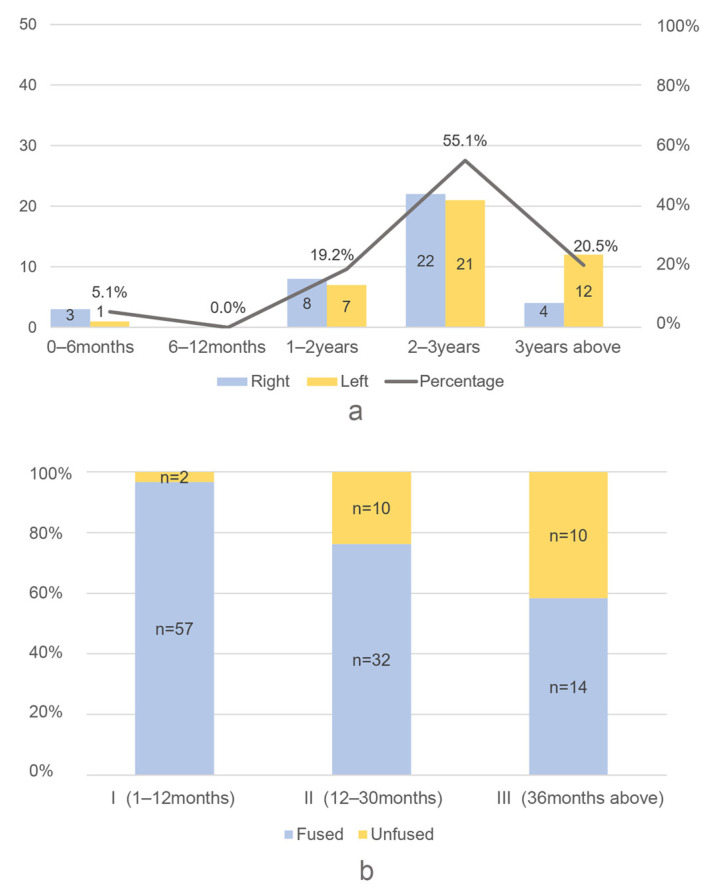
Suid age profile estimated by (**a**) mandible teeth and (**b**) epiphyseal fusing.

The age structures of cervids and suids, the two most abundant taxa in Jiangzhuang, were estimated based on the eruption and wearing of the mandibular teeth. There are 78 suid mandibles used for age estimation, and the results indicate that the majority of the suids were culled during adulthood (Figure 6a). The highest number of mandibles was observed in the 2–3 years age group, representing over 50% of the total. The proportions of mandibles in the 1–2 year age group and 3+ year age groups are both approximately 20%. The 0–6 months age group exhibits the lowest number of mandibles, with no individuals observed in the 6–12 months age group. Epiphyseal fusion pattern is also examined (Figure 6b). Suids are grouped into three categories: 1–12 months, 12–30 months, and 36 months above. All suids survived their first year. The survivorship decreases in their second and third years, and 41.7% of individuals died after reaching 3.5 years old. In general, the suid age profile at Jiangzhuang indicates a preference for older adult individuals.

**Figure 7:**
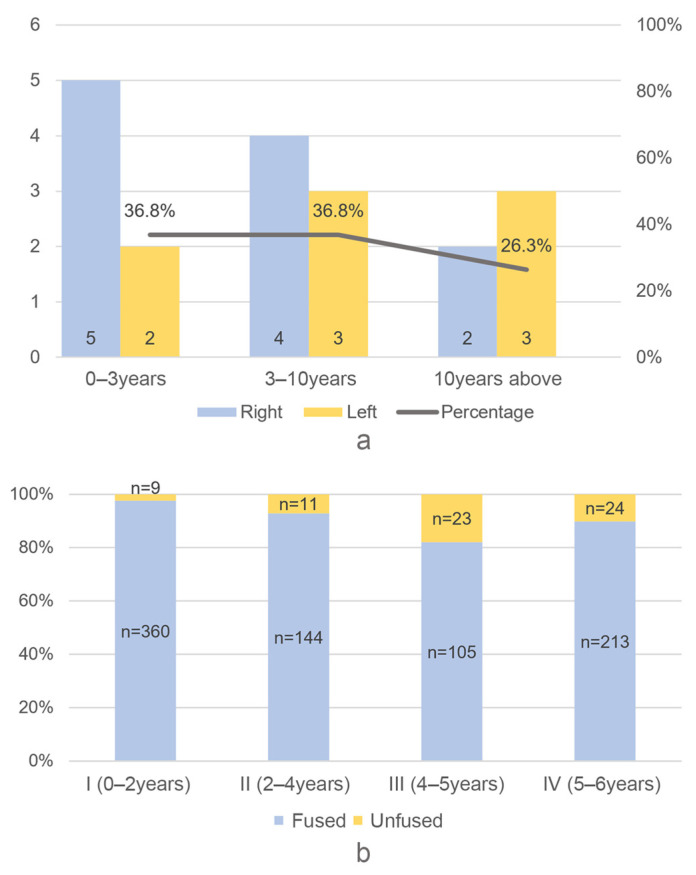
Cervid age profile estimated by (**a**) mandible teeth and (**b**) epiphyseal fusing.

In cervids, 19 mandibles were examined to estimate their age. The discrepancy in the number of left and right specimens is more pronounced than that observed in the suids, which may be due to the small sample size. The tooth eruption and wearing pattern indicate that cervids were culled at different ages, with no obvious accumulation in a particular age group (Figure 7a). In total, 889 bones from different parts of the body were examined for epiphyseal fusion. The survival rate is 97.6% in the 0–2 years age group, 90% in the 2–4 years age group, and 80% in the 4–5 and 5–6 years groups (Figure 7b). In general, a significant number of deer have survived to an older age.

### 3.3. Sex Distribution and Seasonality

A total of 31 crania from milu deer were preserved for sex estimation. Only seven specimens (left: 4; complete: 3) were derived from female individuals. Among the sika assemblage, there are 25 crania (left: 7; right: 13; complete: 5) from males and five females (left: 2; right: 3; complete: 2). The sex distribution is apparently biased to males.

We also examined the seasonality according to the antler cycle. Milu deer normally wear no antlers from December to February [32], and male sika deer usually shed antlers in spring [33,34]. Thirteen milu’s crania wear antlers (left: 5; right: 5; complete: 3) and 14 have burrs indicating naturally shed antlers (left: 5; right: 4; complete: 5). Among sikas, five crania present traces of shed antlers (left: 1; right: 3; complete: 1) and 20 with antlers (left: 6; right: 10; complete: 4). It appears that deer were hunted throughout the year.

### 3.4. Body Element Distribution

**Figure 8:**
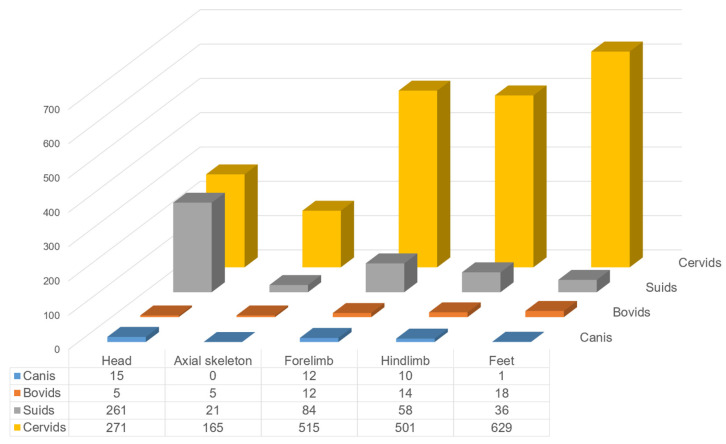
Body element distribution of major animal taxa.

Body element distribution was examined to assess the utilisation of animal body carcasses. Antlers and teeth are excluded, given the possibility that these specimens may have been derived from one individual. Cervids, suids, bovids, and canids were examined, and their body parts were categorised into four skeletal parts: head (skull, maxillary, mandible), axial skeleton (relatively complete vertebrae and ribs), forelimb (scapula, humerus, ulna, and radius), hindlimb (pelvis, femur, patella, tibia, fibula), and foot parts (carpal, metacarpal, calcaneum, astragalus, tarsal, metatarsal, and phalanges) (Figure 8).

The lowest frequency of axial bones is associated with difficulty in identifying vertebrae and ribs. A large number of head parts were recovered from the suids (56.8%) and canids (39.5%). Furthermore, suids (forelimbs = 18.2%, hindlimbs = 12.6%) and canids (forelimbs = 31.6%, hindlimbs = 26.3%) also exhibited a relatively higher presence of limbs and under-representation of feet bones (suids = 7.8%, canis = 2.6%). In contrast, cervids and bovids’ feet bones have the largest proportion (cervids = 30.2%, bovids = 33.3%), followed by limbs (cervids’ forelimbs = 24.8%, hindlimbs = 24.1%; bovids’ forelimbs = 22.2%, hindlimbs = 26.0%). Only a small number of bones belong to the skull (cervids = 13.0%, bovids = 9.3%). The body element distribution tentatively suggests two distinct dismembering and discarding patterns between the cervids and bovids and between suids and canids.

The frequency of bone elements was not calculated for the other animals because of their limited number of identified specimens.

### 3.5. Surface Modification

A variety of agents have left modifications on bones. Faunal remains unearthed at the Jiangzhuang site, which displays evidence of both human and animal modifications. The analysis of human modifications can facilitate an understanding of how ancient people acquired and utilised animal carcasses. Furthermore, the traces left by animals are indicative of taphonomic processes.

**Figure 9:**
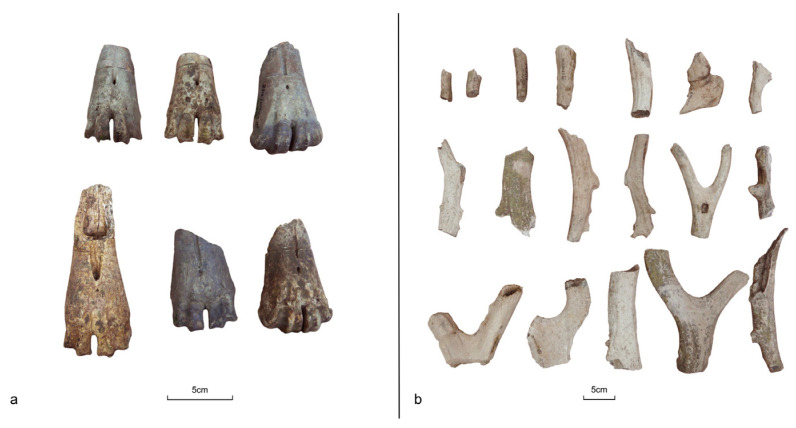
Surface modifications: (**a**) chopping marks on cervid metatarsals; (**b**) worked antlers.

Given that antlers were fragmented and not representative when assessing the quantity in comparison with bones, surface modifications on bones and antlers were analysed, respectively. Among antlers, 22.7% of specimens (NISP = 188) have obvious modifications (Figure 9), where chopping (NISP = 119) and dismemberment cutting (NISP = 65) are relatively frequent.

For bones, most human modifications also occurred on cervids (n = 202), whereas a limited number of modifications were found in suids (n = 14) and bovids (n = 1) (Table 2). Four distinct types of human modification are recognised at the Jiangzhuang site: chopping, dismemberment cutting, polishing, and burning. Chopping and cutting, which are related to the processes of butchering and slaughter, account for over 50%. We further examined the distribution of surface modifications in cervids to obtain more detailed information on cervid exploitation.

**Figure 10:**
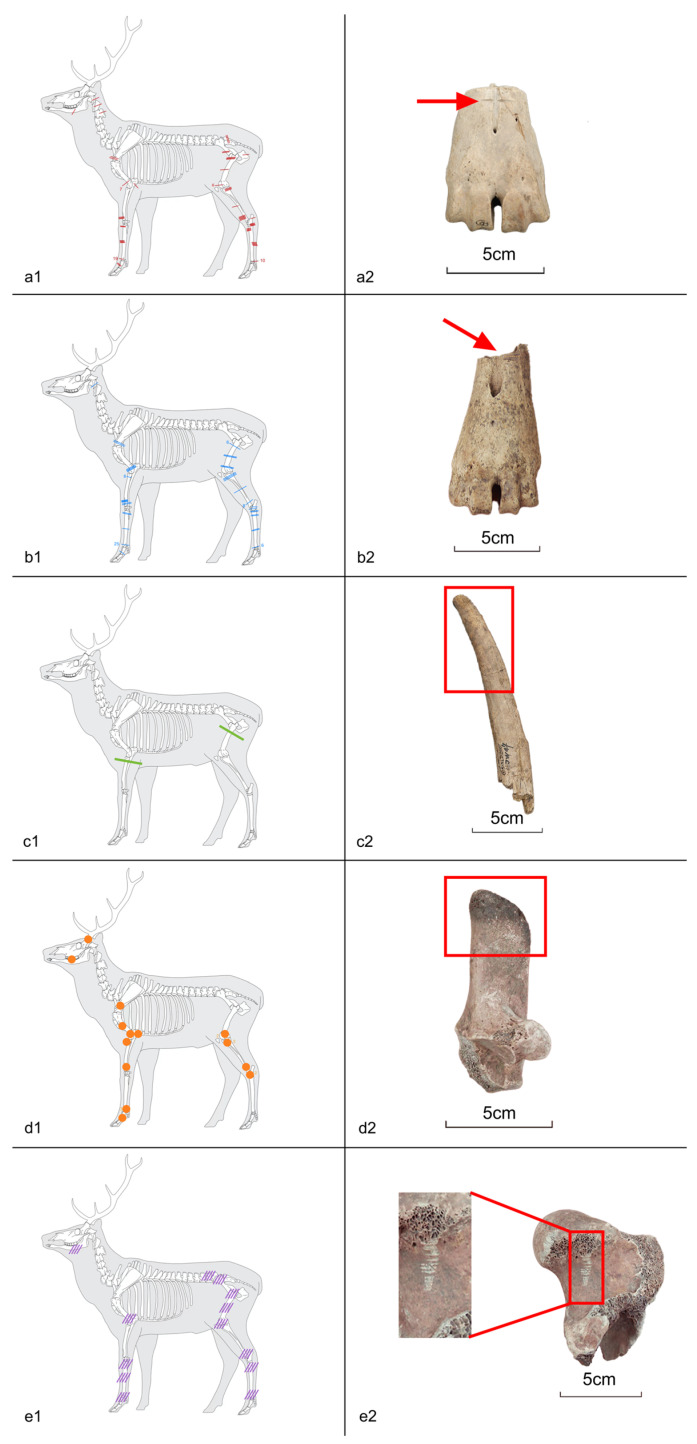
Distribution of bone surface modifications on cervids: (**a1**) distribution of chopping marks; (**a2**) metacarpal with chopping mark on; (**b1**) distribution of dismemberment marks; (**b2**) metatarsal with dismemberment mark; (**c1**) distribution of polishing marks; (**c2**) antler with polishing mark; (**d1**) distribution of burning marks; (**d2**) calcaneus with burning mark; (**e1**) distribution of gnawing marks; (**e2**) femur gnawed by rodents (illustrations modified from J.-G. Ferrié © ArcheoZoo. org [44]).

The percentage of surface modifications found in cervids is 9.2%. The chopping marks (NISP = 94) were mainly concentrated on limb bones (Figure 10(a1,a2)). The second most abundant body part with surface modifications is the metapodium, with a higher occurrence on hindlimbs. Besides, some chopping marks were recognised on the mandibles, pelvis, cervical vertebrae, and anterior part of the caudal vertebrae.

The distribution of dismemberment cutting marks (NISP = 84) is similar to that of chopping marks in cervids, where the majority of the marks occur at the metapodium, followed by the forelimbs and hindlimbs (Figure 10(b1,b2)). The posterior part of the occipital bone, which was connected to the cervical vertebrae, was also cut off.

It can be seen that Jiangzhuang residents used chopping and dismemberment to separate the head and the limbs from the axis skeleton. There are two types of breaking methods based on the bone fracture shape: complete separation by cutting and two-step separation by cutting around the bone shaft and then manually breaking the bones along the cut marks.

Two polishing traces occur at the proximal end of the radius and femur, respectively, likely associated with tool manufacturing (Figure 10(c1,c2)).

Burning marks (NISP = 22) were found in several body elements, including crania, teeth, limbs, and metapodium (Figure 10(d1,d2)). Burnt specimens are brownish-black, and no fully calcined specimens were found. However, it remains unclear whether they were left by humans or natural agents.

Rodent gnawing (NISP = 20) and carnivore biting (NISP = 1) also left marks on the cervid bones from Jiangzhuang. While limb joints and diaphyses of metacarpals and femurs were most frequently gnawed by rodents, only one cervid distal tibia was pierced, probably by a carnivore’s canine (Figure 10(e1,e2)).

## 4. Discussion

### 4.1. Low-Level Food Production at Jiangzhuang

The faunal remains from Jiangzhuang are largely refuse in daily life, as indicated by their context and taphonomic processes. Therefore, they are informative about the past lifeway. Wild animals accounted for the largest proportion of the faunal assemblage, suggesting that hunting was practised. Cervids, the most abundant taxa in the assemblage, were likely the primary food resources of the Jiangzhuang residents. In addition, tigers, alligators, softshell turtles, and several birds were present in the faunal assemblage. These species live in diverse habitats. Their presence demonstrates that Jiangzhuang residents lived in the vicinity of a wide range of environments, such as forests, swamps, and lakes, where diverse animal resources were easily accessible.

Suids also comprise a large proportion of the faunal assemblage. Morphological characteristics highlight the co-existence of multiple types of suids, including domesticated pigs, wild boars, feral pigs, and probably hybrids of the three. The mixture of suid populations is further supported by the molar size metric data, which do not exhibit an obvious polarised distribution. Domesticated pigs were maintained on a small scale, the specific way of husbandry, however, remains unclear. Future isotope analyses may decipher whether they were pen-herded in an intensive or free-range form. At the same time, wild boars were targeted prey. Seemingly, the Neolithic Jiangzhuang residents were both hunters and herders.

The exploitation of plant resources demonstrates a similar pattern. Previous archaeobotanical results indicate that the Jiangzhuang residents were both farmers and foragers. The predominant presence of rice, together with sedges (a type of weed that infests rice paddy fields), is evident for rice cultivation. Small-scale horticulture produced bottle gourds and melons. Meanwhile, a variety of wild plants were gathered, including fruits growing in forests such as peaches, plums, dates, and kaki fruits, and wetland staple foods such as water chestnut and lotus root [45]. The habitats where these plants grow overlap with those of cervids and wild boars.

This way of life at Jiangzhuang persisted in the form of low-level food production, where farming and husbandry were never pursued on a scale large enough to disrupt pre-existing hunter-gatherer subsistence patterns. It seems that the low regional population densities at Jiangzhuang, along with the rich wild game and plants in the surrounding environments, made the reliance on wild resources less costly than full-time agriculture.

Previous studies have revealed that wild resources have been extensively utilised in the Jianghuai region since the early Neolithic [46], as reflected in several sites such as Longqiuzhuang [5], Qingdun [47] (pp. 87–92), and Kaizhuang [19]. In conjunction with paleoenvironmental studies, this region is characterised by a dense network of rivers and an abundance of marshy wetlands. To this day, this area remains a significant reserve for the protection of milu deer. The richness of wild resources might have delayed agricultural intensification. In fact, horticulture, often involving the intercropping of a wide range of plants, manifests that this plant production somehow mirrored the foraging strategy incorporating extensive exploitation of various wild resources. Rice cultivation, to some degree, was also a plant management strategy parallel to wetland exploitation. Correspondingly, pigs, which easily adapt to various environments and require less care, fit well into this low-level food production. Free-ranging seemed a less labour-costly way to manage pigs. As a result, the loose management of pigs might have increased the possibility of hybrids with wild boars and the existence of feral pigs, in agreement with the ambiguous morphology of suid remains.

At the same time, increasing frequencies of marine invasions and coastal flooding during the late Liangzhu period might have obscured the ongoing development of agriculture [48].

In addition to environmental factors, social factors might also have influenced subsistence choices. Given the injuries frequently found in human skeletons, Jiangzhuang is considered a military front combating other cultural groups [21,22]. This hypothesis renders a tentative explanation for subsistence strategy. In contrast to the arduous and time-consuming nature of animal husbandry, hunting demands less effort, especially when wild games are available nearby. Consequently, hunting proved to be a more practical strategy when the military need for labour escalated.

### 4.2. Intensive Exploitation of Cervids

The use of cervids might have been intensive. First and foremost, cervids were the primary food resources. Cervids of various body sizes are present in the assemblage. The estimation of cervid age indicates that cervids in a wide range of age groups were hunted. The sex distribution is slightly biased towards males, probably because antlers were desirable. This selective strategy primarily aimed at stags and avoiding breeding females appeared more sustainable, contributing to the long-term stability of the cervid population. The presence or absence of antlers on the cranium suggests that deer were hunted throughout the year. The body element distribution, where feet bones have a large proportion, suggests that cervids were transported back to the site in the whole carcass, and even those body parts with less meat were also utilised. This cervid exploitation strategy seemingly has ensured sufficient food provision.

Bones and antlers, in addition to meat, were also important resources provided by cervids at Jiangzhuang. Naturally-shed antlers were collected and modified for tool production. Stags that were hunted during the seasons when they bore antlers were culled not only for meat but also for antlers and bones. The prevalence of cervid feet bones, which serve as an ideal raw material for bone tools, is also associated with tool production. Consequently, cervid bones and antlers bear the most human modifications. Chopping and dismemberment cutting marks were distributed mainly around the joints, which would cut the carcass into large chunks for cooking and consumption. Burning traces were also found around the joints, which might have resulted from roasting, as the fire directly charred the ends of the bones that had minimal soft tissue attached. Some of the bones might have been deposited in the open air for some time, during which rodents and dogs came and gnawed the remaining meat on the bones.

After consumption, the remaining bones were further utilised to make tools. Bone awls, fish hookers, and arrowheads were recovered at the site. They were made from long bones of large herbivores, which were very likely cervid bones, considering their shape and strength. Large deer metapodia were frequently used in tool production. This may explain the high frequency of cervid metapodia in Jiangzhuang. The shaft of the metapodium, which is straight and has a thick bone wall, was cut from the joints, leaving conspicuous cut marks around the nutrient foramen at the distal end. The examination of cutting marks displays two types of breaking: one is to cut the bone shaft thoroughly off the joints, and the other is to cut first and then split the bone shaft manually. Most cervid metapodium was produced in the first way, indicating that they were carefully prepared to maximise the usage of the bone. Given the identical location of these cutmarks on metapodia, it is likely that a streamlined tool production industry had formed at Jiangzhuang. Similar tool production pattern is seen in Huanggang [49] and Longqiuzhuang as well [50]. More evidence is needed to depict the bone tool industry in detail.

Apart from bones, antlers were also ideal raw materials for tool manufacturing. A significant number of antlers were recovered from the site, 22.7% of which had undergone surface modifications. Both unshed and shed antlers were used. Shed antlers were easily collected from surrounding forests and were frequently used for tool production. Further studies focusing on tool production will help unveil the diverse exploitation of animal products.

### 4.3. Economic Independence in Liangzhu Peripheries

The eastern region of the Lower Yangtze and the Huai River, also called the ‘Jianghuai’ region, where the Jiangzhuang site is located, was a central avenue of cultural interaction during the Neolithic period. Before the Liangzhu period, the Qingliangang (ca. 5400–4400 BC), Majiabang (ca. 5000–4000 BC), and Luotuodun (ca. 5000–3000 BC) cultures had bridged the Jianghuai region and the Lake Tai Basin [20]. After the remarkable growth of cultural interactions during the Songze period (ca. 3800–3300 BC), the Liangzhu culture arrived in the Jianghuai region along the eastern coast. While retaining a strong local tradition, the material culture of Jiangzhuang also absorbed a significant number of Liangzhu cultural elements. Jade artefacts, in particular, were nearly identical to those from the Liangzhu core area, reflecting the growing influences from the Liangzhu culture moving northward. The Liangzhu influence, however, seemed restricted to jade, the material manifesto of Liangzhu ideology. Objects for daily conduct largely remained consistent in local style. Therefore, Jiangzhuang was more likely a local node within the hierarchical social network of the Lianbgzhu society, where the Liangzhu ancient city was at the top of the social pyramid [20]. At this rural periphery, the Liangzhu institutional groups would not have bothered with the local subsistence as long as their ideological connection was firmly forged. Consequently, the subsistence economy at Jiangzhuang, fully taking advantage of the local environment, was independent of the Liangzhu core area’s agricultural production and urban lifeway.

Previous studies in Liangzhu peripheral areas have demonstrated a comparable pattern where local settlements were tenuously affiliated with the Liangzhu core area’s subsistence economy despite the conspicuous Liangzhu element exhibited in jade styles. Very likely, Liangzhu’s control primarily focused on the superstructure, facilitated by a shared religious cult among the upper echelons of the society. Meanwhile, the economic foundations of these remote local communities have remained largely unregulated. As a consequence, Jiangzhuang had long relied primarily on wild resources, supplemented by a modest level of food production.

Liangzhu’s mode of control deviates from its neighbouring culture. In the north Jianghuai region, the Liangzhu culture encountered the Xuejiagang and Dawenkou cultures, with prominent exchanges manifested in the material culture [51]. While the Jiangzhuang residents maintained a low level food production, the farming lifeway had been widely adopted by the surrounding cultural groups. The faunal assemblages recovered from several Dawenkou culture sites in the north Jianghuai region, such as Jianxin and Dawenkou, include a major proportion of domesticated animals [52]. Archaeobotanical studies at Yangpu [53] and Gongzhuang [54] also reveal a mixed farming model of rice and millet. Unlike Liangzhu’s mode of control, the influences from these cultures seemed more pervasive, and the local economy was more or less uniform. Jiangzhuang, however, rejected a total shift to intensive agriculture that had been practised over a long time by surrounding cultural groups. Owing to this distinctive mode of control, the economic independence in the peripheral Liangzhu areas might have contributed to the increasing decentralisation in the late Liangzhu period, providing a plausible explanation for the collapse of the Liangzhu society.

## 5. Conclusions

At the northern frontier of Liangzhu, Jiangzhuang developed a local way of life by taking advantage of its surrounding environment. The faunal assemblage is primarily composed of cervids with a small proportion of livestock, indicating subsistence heavily relying on wild resources. Not only were domesticated pigs raised at a low level, but the presence of morphologically domesticated pigs, wild boars, and those in between adds another layer of complexity to suid exploitation, probably involving hunting, free-ranging, feralisation, crossbreeding, and other management strategies. The intensive utilisation of cervid bones and antlers for tool production is also a distinctive feature. While the Jiangzhuang community might have been articulated with the Liangzhu centre through the circulation of ritual jades, the subsistence economy there remained largely self-sufficient. From a wider perspective, religious fanaticism, military conflicts, and natural disasters together might have contributed to the collapse of the Liangzhu civilisation. The economic autonomy in Liangzhu’s peripheral areas might have also played a role in the increased decentralisation observed in its final phase, offering a subsistence explanation for the social changes in the late Liangzhu period.

## Figures and Tables

**Table 1:** NISP of animal taxa and their relative proportion at Jiangzhuang (note: percentage lower than 1.0% is not listed).

Taxon	NISP	NISP%
Reptile		
*Alligator sinensis*	1	
*Pelochelys cantorii*	1	
Testudines	61	1.5%
Bird		
*Anser* sp.	5	
Anatidae	1	
*Cygnus* sp.	1	
Gruidae	2	
Unknown species	4	
Mammal		
Rodentia	2	
*Canis lupus familiari*	39	1.0%
*Panthera tigris*	1	
Viverridae	1	
Suid	536	13.9%
Cervid *	3026	76.7%
Bovid	61	1.6%
Unknown species	203	5.0%
Total	3945	

* including antlers (n = 827).

**Table 2:** Summary of surface modifications.

	Chopping	Dismemberment Cutting	Polishing	Burning
Cervid	94	84	2	22
Suid	11	1	1	1
Bovid	1	0	0	0
Total	106	85	3	23

## Data Availability

All relevant data are included in this manuscript. The faunal assemblage list will be made available by the author on request.

## Supplementary Materials

The following supporting information can be downloaded at: https://www.mdpi.com/article/10.3390/ani14233461/s1, Table S1: Measurements of the mandible third molar from suids.
