# Supplementary material for: Herding or Hunting? Animal Exploitation at Jiangzhuang (Jiangsu, China) During the Liangzhu Period (3300–2300 BC)
Source: Animals (Basel). 2024 Nov 29;14(23):3461. doi: 10.3390/ani14233461 (PMC11640202; doi:10.3390/ani14233461)
Supplement: Supplementary file 1 [file animals-14-03461-s001.zip › animals-3328361-supplementary.pdf]

Supplementary materials

Table S1. Measurements of the mandible third molar from suids.

Context	Taxa	Side	Length	Breadth
2011JWIT515(12)	*Sus scrofa*	left	32.18	16.49
2012JWTG2(20)	*Sus scrofa*	left	39.01	17.6
2012JWTG2(20)	*Sus scrofa*	left	37.26	15.57
2012JWTG2(20)	*Sus scrofa*	left	36.43	16.43
2011JWIT518(7)	*Sus scrofa*	right	41.62	17.46
2011JWIT113(7)	*Sus scrofa*	left	38.74	15.62
2012JWTG2(18)扩	*Sus scrofa*	right	38.74	17.66
2012JWTG2(18)扩	*Sus scrofa*	left	35.49	17.05
2012JWTG2(18)扩	*Sus scrofa*	left	33.69	15.42
2011JWIT515(8)	*Sus scrofa*	left	38.61	17.01
2011JWIT519(5)	*Sus scrofa*	right	39.3	17.08
2012JWM97	*Sus scrofa*	left	34.76	15.78
2012JWTG3(13)	*Sus scrofa*	right	41.93	16.75
2012JWITG3(9)	*Sus scrofa*	left	31.86	15.51
2012JWITG3(11)	*Sus scrofa*	left	41.24	17.42
2011JWIT212(10)	*Sus scrofa*	right	41.04	18.68
2012JWTG2(19)	*Sus scrofa*	left	33.25	15.54
2015JWG19	*Sus scrofa*	left	38.17	16.67
2012JWITG3 西扩(12)	*Sus scrofa*	left	38.07	16.25
2012JWITG3 西扩(12)	*Sus scrofa*	left	42.08	17.84
2012JWJ13	*Sus scrofa*	left	44.92	18.22

2015JWTG21	*Sus scrofa*	right	38.05	16.63
2015JWTG21	*Sus scrofa*	left	40.09	17.77
2015JWTH162	*Sus scrofa domesticus*	right	34.19	16.66
2016JWT6(11)	*Sus scrofa*	right	39.12	15.6
